# Calcium-dependent binding of Myc to calmodulin

**DOI:** 10.18632/oncotarget.13759

**Published:** 2016-12-01

**Authors:** Philipp Raffeiner, Andrea Schraffl, Thomas Schwarz, Ruth Röck, Karin Ledolter, Markus Hartl, Robert Konrat, Eduard Stefan, Klaus Bister

**Affiliations:** ^1^ Institute of Biochemistry and Center for Molecular Biosciences, University of Innsbruck, A-6020 Innsbruck, Austria; ^2^ Max F. Perutz Laboratories, Department of Structural and Computational Biology, University of Vienna, A-1030 Vienna, Austria; ^3^ Present address: Department of Molecular and Experimental Medicine, The Scripps Research Institute, La Jolla, CA 92037, USA

**Keywords:** oncogene, transcription factor, signal transduction, protein-protein interaction, second messenger signaling

## Abstract

The bHLH-LZ (basic region/helix-loop-helix/leucine zipper) oncoprotein Myc and the bHLH-LZ protein Max form a binary transcription factor complex controlling fundamental cellular processes. Deregulated Myc expression leads to neoplastic transformation and is a hallmark of most human cancers. The dynamics of Myc transcription factor activity are post-translationally coordinated by defined protein-protein interactions. Here, we present evidence for a second messenger controlled physical interaction between the Ca^2+^ sensor calmodulin (CaM) and all Myc variants (v-Myc, c-Myc, N-Myc, and L-Myc). The predominantly cytoplasmic Myc:CaM interaction is Ca^2+^-dependent, and the binding site maps to the conserved bHLH domain of Myc. Ca^2+^-loaded CaM binds the monomeric and intrinsically disordered Myc protein with high affinity, whereas Myc:Max heterodimers show less, and Max homodimers no affinity for CaM. NMR spectroscopic analyses using alternating mixtures of ^15^N-labeled and unlabeled preparations of CaM and a monomeric Myc fragment containing the bHLH-LZ domain corroborate the biochemical results on the Myc:CaM interaction and confirm the interaction site mapping. In electrophoretic mobility shift assays, addition of CaM does not affect high-affinity DNA-binding of Myc:Max heterodimers. However, cell-based reporter analyses and cell transformation assays suggest that increasing CaM levels enhance Myc transcriptional and oncogenic activities. Our results point to a possible involvement of Ca^2+^ sensing CaM in the fine-tuning of Myc function.

## INTRODUCTION

The *myc* oncogene was originally discovered as the oncogenic principle (v-*myc*) in the genome of avian acute leukemia virus MC29, derived from the chicken proto-oncogene c-*myc* by retroviral transduction [1–3]. The discovery of chromosomal translocations of the human *MYC* gene in Burkitt lymphoma cells provided the first connection of the proto-oncogenic homolog of a retroviral oncogene with human carcinogenesis [4]. Today, deregulated *MYC* expression is established as an important driving force in the majority of all human cancers [1, 2, 5, 6]. The Myc protein, originally identified as a Gag-Myc hybrid protein (p110) specified by MC29 genomic RNA [3, 7], is a transcriptional regulator of the basic/helix-loop-helix/leucine zipper (bHLH-LZ) protein family, forms binary complexes with the bHLH-LZ protein Max, binds to specific DNA sequence motifs (E-box), and is the central hub of a ubiquitous transcription factor network [8–10]. In human cells, dynamic Myc:Max network interactions control thousands of genes involved in fundamental cellular processes like cell growth, proliferation, biosynthesis, energy metabolism, differentiation, and apoptosis [5, 6, 9, 10].

Myc:Max heterodimers usually function as transcriptional activators of target genes, but Myc can also be involved in transcriptional repression [5, 6, 9, 10]. We have previously described the identification of a Myc target gene (*BASP1*, also termed *CAP-23*/*NAP-22*) that is repressed in Myc-transformed cells and, when expressed ectopically, inhibits cell transformation by Myc [11]. *BASP1* encodes a small acidic protein that was originally isolated as a membrane and cytoskeleton-associated protein from brain [12], but was also found as a nuclear cofactor of the Wilms’ tumor suppressor WT1 [13]. The BASP1 protein is a substrate of protein kinase C and N-myristoyltransferase, and binds tightly to calmodulin (CaM) [12, 14]. CaM is a small highly conserved EF-hand protein in eukaryotes that functions as the major intracellular receptor for the second messenger Ca^2+^. Ca^2+^-dependent signaling pathways control fundamental cellular processes, and a large number of target proteins, e.g. kinases, phosphatases, ion channels, and others, are bound by CaM and modulated in their function [15, 16]. Interestingly, it has been reported that CaM can also bind to and modulate the activity of transcriptional regulators of the bHLH class like E12, E47, or SEF2-1 [17–19]. In view of this and emanating from the identification of the Myc target *BASP1* encoding a CaM-binding protein, we searched for a possible connection of the bHLH-LZ proteins Myc and Max with CaM, and analyzed the observed interactions in structural and functional detail.

## RESULTS

### Detection of Myc:CaM interaction in GST-CaM and CaM-agarose pull-down assays

**Figure 1:**
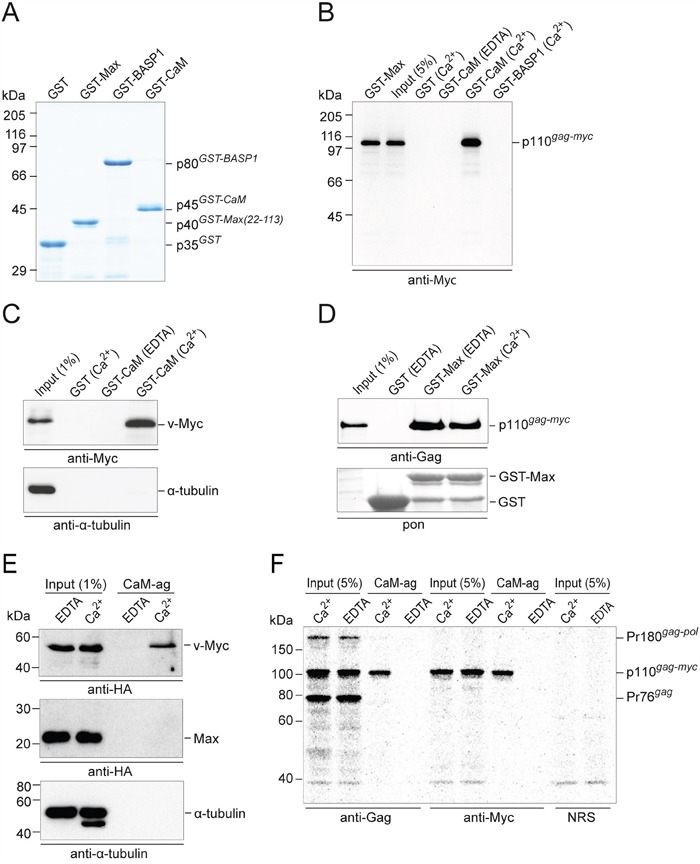
Ca^2+^-dependent PPI of Myc and CaM **A.** SDS-PAGE (12% wt/vol) and Coomassie brilliant blue staining of purified recombinant GST-fusion proteins used in pull-down experiments. **B.** Pull-down assay with GST-fusion proteins and whole cell extracts prepared from QEF/MC29 cells metabolically labeled with [^35^S]-methionine. These cells express the p110 Gag-Myc hybrid protein. After protein pull-down, protein complexes were eluted from the glutathione sepharose beads for immunoprecipitation under denaturing conditions using Myc-specific antiserum. Following SDS-PAGE, protein bands were visualized by fluorography. **C.** Pull-down experiments using GST-fusion proteins and QEF/Rc-Myc cell lysates containing the 52-kDa v-Myc protein. Proteins eluted from the glutathione-sepharose beads were subjected to SDS-PAGE and immunoblotting using antibodies directed against Myc or α-tubulin. **D.** Myc:Max complex formation was analyzed in pull-down experiments using GST-Max as bait protein and whole cell extracts from Q8 cells. The Gag-Myc hybrid protein was detected by immunoblotting using a Gag-specific antiserum. A section of the membrane with Ponceau S-stained GST bait proteins is shown (pon). EDTA, 2 mM; Ca^2+^, CaCl_2_ 0.5 mM. **E.** QEF/RCAS-HA-v-Myc cells were used for the analysis of the 53-kDa HA-tagged v-Myc protein using CaM-agarose (CaM-ag) as affinity matrix. For comparison, overexpressed HA-tagged Max protein and endogenous α-tubulin were analyzed in extracts from QT6 cells. **F.** Specific pull-down of the Gag-Myc hybrid protein bound to CaM-ag. Lysates prepared under native conditions from metabolically [^35^S]-methionine-labeled QEF/MC29 cells were loaded onto CaM-ag beads in the presence of CaCl_2_ (0.5 mM) or EDTA (1 mM). Bound proteins were eluted under denaturing conditions, and subjected to immunoprecipitation using polyclonal antibodies directed against Gag or Myc proteins, or normal rabbit serum (NRS). As input control, 5% of the lysates were used for immunoprecipitation. Proteins were resolved by SDS-PAGE (10% w/v) and detected on a bioimager. Positions of protein size markers are indicated in the margin.

For the initial analyses of possible protein-protein interactions (PPIs) between Myc and CaM, recombinant glutathione *S*-transferase(GST)-fusion proteins containing chicken Max, CaM, or BASP1 as bait segments were synthesized (Figure 1A). These GST-fusion proteins were used in pull-down assays performed with whole cell extracts from quail embryo fibroblasts (QEF) transformed by MC29. As expected, the MC29-encoded 110-kDa Gag-Myc hybrid protein [7] coprecipitated with GST-Max, but with similar efficiency also with GST-CaM (Figure 1B). No complex formation of p110 was observed with GST alone, or with GST-BASP1, confirming previous results that Myc and BASP1 do not interact directly [11]. Notably, the Gag-Myc:CaM interaction was strictly Ca^2+^-dependent and completely abolished upon addition of the chelating agent ethylenediaminetetraacetic acid (EDTA) (Figure 1B). Efficient Ca^2+^-dependent pull-downs of a 52-kDa v-Myc protein devoid of Gag from extracts of QEF/Rc-Myc cells [11] strongly suggested that the p110:CaM interaction is due to the Myc moiety of the Gag-Myc hybrid protein, and endogenous α-tubulin used as a negative control did not interact with GST-CaM in the pull-down analyses (Figure 1C). The specificity of the stringent Ca^2+^-dependence of the Myc:CaM interaction (Figure 1B and 1C) was corroborated by the pull-down of the Gag-Myc hybrid protein by GST-Max with equal efficiency relative to input both in the presence or absence of Ca^2+^ (Figure 1D). As an independent method, binding of Myc proteins to CaM was also analyzed by pull-down on CaM-agarose as affinity matrix. The 53-kDa HA-tagged v-Myc protein expressed in QEF transformed by a HA-v-Myc construct showed Ca^2+^-dependent binding to CaM with similar efficiency as in the GST pull-downs (Figure 1E). Overexpressed HA-tagged Max protein or endogenous α-tubulin from extracts of QT6 cells did not bind to CaM (Figure 1E). Analysis of extracts from QEF/MC29 cells transformed by a replication-defective RCAS-MC29 construct in the presence of replication-competent RCAS helper virus underscored the high specificity of the Myc:CaM interaction and the stringent Ca^2+^-dependence. Immunoblots using anti-Gag serum detected all Gag-containing proteins in the input: the Pr180 Gag-Pol precursor and the Pr76 Gag precursor of the RCAS helper virus, and the p110 Gag-Myc hybrid protein of MC29. However, in the CaM-agarose pull-down, only the Myc containing hybrid protein was detected, but not the Gag-containing structural protein precursors (Figure 1F). Using anti-Myc serum, both input and pull-down showed the p110 protein only (Figure 1F). Hence, the Myc domain, but not the Gag domain of the Gag-Myc hybrid protein is responsible for the CaM interaction.

### Mapping the CaM binding region of Myc

**Figure 2:**
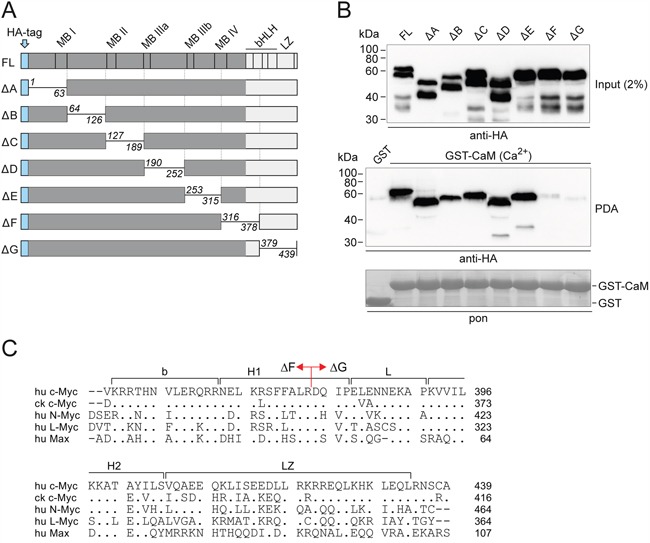
Mapping of the Myc:CaM interaction domain **A.** Schematic depiction of full-length (FL) human c-Myc and scanning deletion (Δ) mutants. The mutant proteins lack residues 1-63 (A), 64-126 (B), 127-189 (C), 190-252 (D), 253-315 (E), 316-378 (F), and 379-439 (G), respectively [20, 21]. bHLH, basic region/helix-loop-helix; LZ, leucine zipper; MB, Myc box; HA, hemagglutinin. **B.** Full-length c-Myc and the deletion mutants were expressed in QT6 cells. Whole cell lysates were used for pull-down experiments with GST-CaM in the presence of 0.5 mM CaCl_2_ (Ca^2+^), and with GST as negative control. An aliquot of the cell lysate (input, *upper panel*) and eluted proteins from the pull-down assay (PDA, *middle panel*) were subjected to SDS-PAGE and immunoblotting using HA-specific antibodies. A section of the membrane with Ponceau S-stained GST bait proteins is shown (pon, *lower panel*). **C.** Alignment of the amino acid sequences of the bHLH-LZ domains of human (hu) c-Myc, chicken (ck) c-Myc, and human L-Myc, N-Myc, and Max. Identities with the human c-Myc sequence used as reference are indicated by dots, gaps are marked by dashes. On the human c-Myc sequence, the border between the ΔF and ΔG deletions (cf. panel A) is indicated.

In order to map the region of the Myc protein necessary for CaM binding, we used a set of scanning deletion mutants of the human c-Myc protein (Figure 2A) [20, 21]. QT6 cells were transfected with expression constructs encoding full-length c-Myc or the indicated deletion mutants. Whole cell lysates were used for pull-down experiments with GST-CaM in the presence of Ca^2+^. All mutant proteins were pulled down with equal efficiency as full-length Myc, except mutants ΔF and ΔG, lacking amino acid residues 316-378 and 379-439, respectively (Figure 2B). This maps the essential region for CaM binding to the bHLH-LZ domain relevant for heterodimerization, possibly to the N-terminal moiety around the border between the ΔF and ΔG deletions (cf. Figure 2A, 2C). The experiment provided an additional, independent confirmation of this conclusion. For full-length Myc and most deletion mutants, the input analyses revealed the presence of proteolytic cleavage products (Myc-nick) that are generated by calcium-dependent cytoplasmic proteases and lack the carboxyl-terminal region of Myc beyond residue 298 [21]. None of these products were pulled down by CaM (Figure 2B).

**Figure 3:**
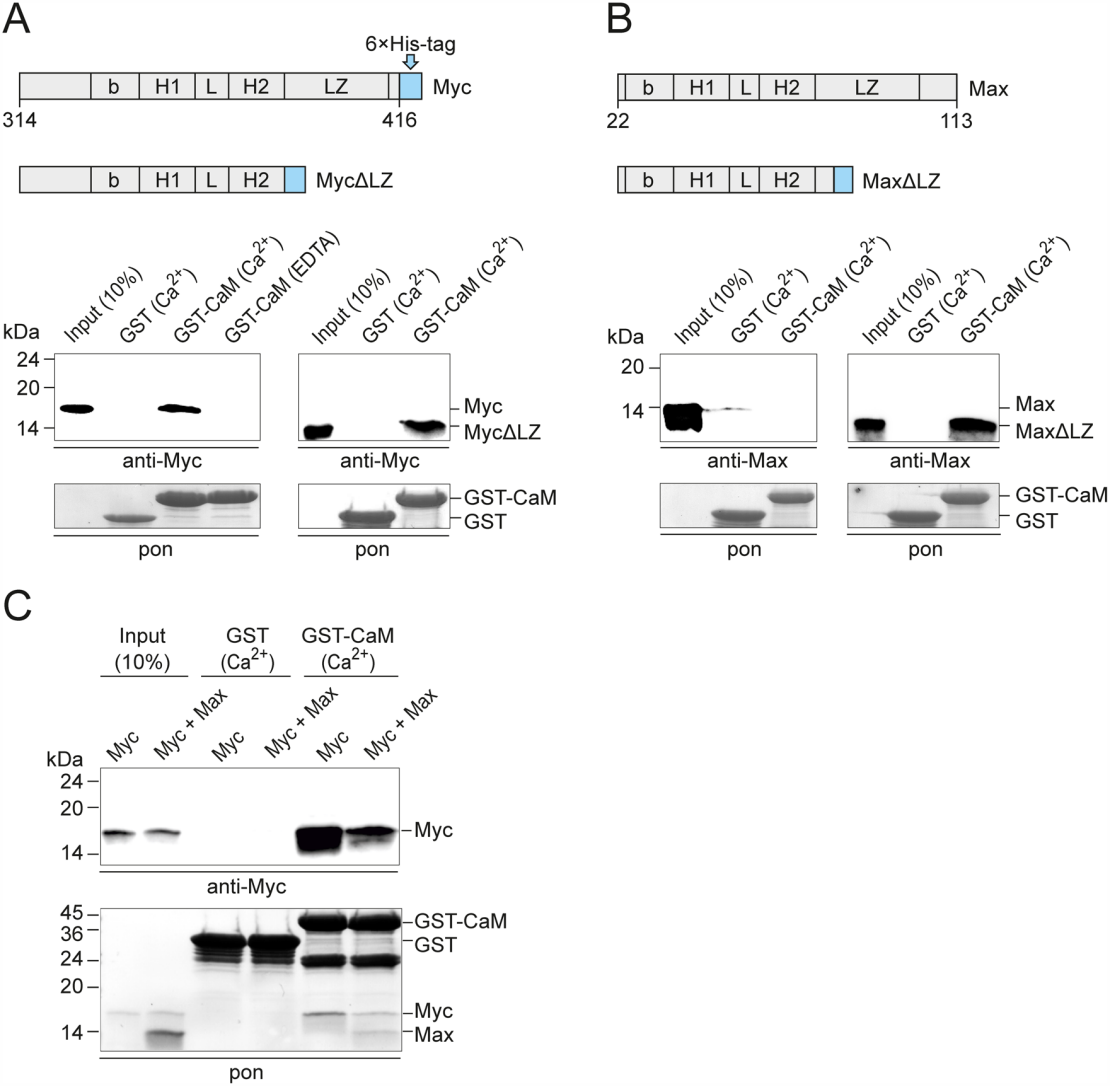
PPI of CaM and bHLH domains of recombinant Myc and Max **A.** GST pull-down experiments using recombinant v-Myc^314-416^ protein or v-Myc^314-383^ lacking the leucine zipper (MycΔLZ) and GST-CaM. Myc proteins were detected by immunoblotting using a Myc-specific antiserum. A section of the membrane with Ponceau S-stained GST bait proteins is shown (pon, *lower panel*). EDTA, 2 mM; Ca^2+^, CaCl_2_ 0.5 mM. **B.** PPI analyses of recombinant Max^22-113^ protein or Max^22-79^ lacking the leucine zipper (MaxΔLZ) and GST-CaM. Max proteins were detected using a Max-specific antiserum. **C.** GST pull-down experiments using recombinant v-Myc^314-416^, Max^22-113^, and GST-CaM in the presence of 0.5 mM CaCl_2_. Where indicated, Myc and Max proteins were mixed using a threefold excess of Max protein. After SDS-PAGE and electroblotting, bait and prey proteins were visualized by Ponceau S-staining (pon, *lower panel*). Myc was detected by immunoblotting using a Myc-specific antiserum (*upper panel*).

For further analyses of the binding region, we used recombinant proteins of Myc and Max encompassing the bHLH-LZ domain and deletion constructs lacking the LZ motif (ΔLZ). In confirmation and extension of the mapping described above, the pull-down assays showed that the Myc protein containing bHLH-LZ, but also MycΔLZ containing bHLH only bound to CaM with equal efficiency (Figure 3A). Interestingly, Max containing the entire bHLH-LZ domain did not bind to CaM, but the construct lacking the LZ motif bound with similar efficiency as Myc (Figure 3B). It is important to note, that recombinant Myc proteins are intrinsically disordered monomers in solution, whereas intact Max readily forms homodimers [22–25]. Also, mutational and structural analyses had revealed that the N-terminal half of the LZ motif of Max is essential for dimerization [23]. Hence, the MaxΔLZ construct lacking the entire LZ motif cannot form stable homodimers and showed CaM-binding properties like the intrinsically monomeric Myc protein. When a mixture of Myc and excess Max recombinant proteins was used, equimolar amounts of Myc and Max were pulled down, apparently by binding of CaM to the Myc:Max heterodimer, albeit with lower efficiency than binding to monomeric Myc (Figure 3C). The PPI experiments using purified recombinant proteins (Figure 3) provided strong evidence for direct interactions independent of other cellular proteins.

### Binding of v-Myc, N-Myc, L-Myc, and c-Myc to CaM

**Figure 4:**
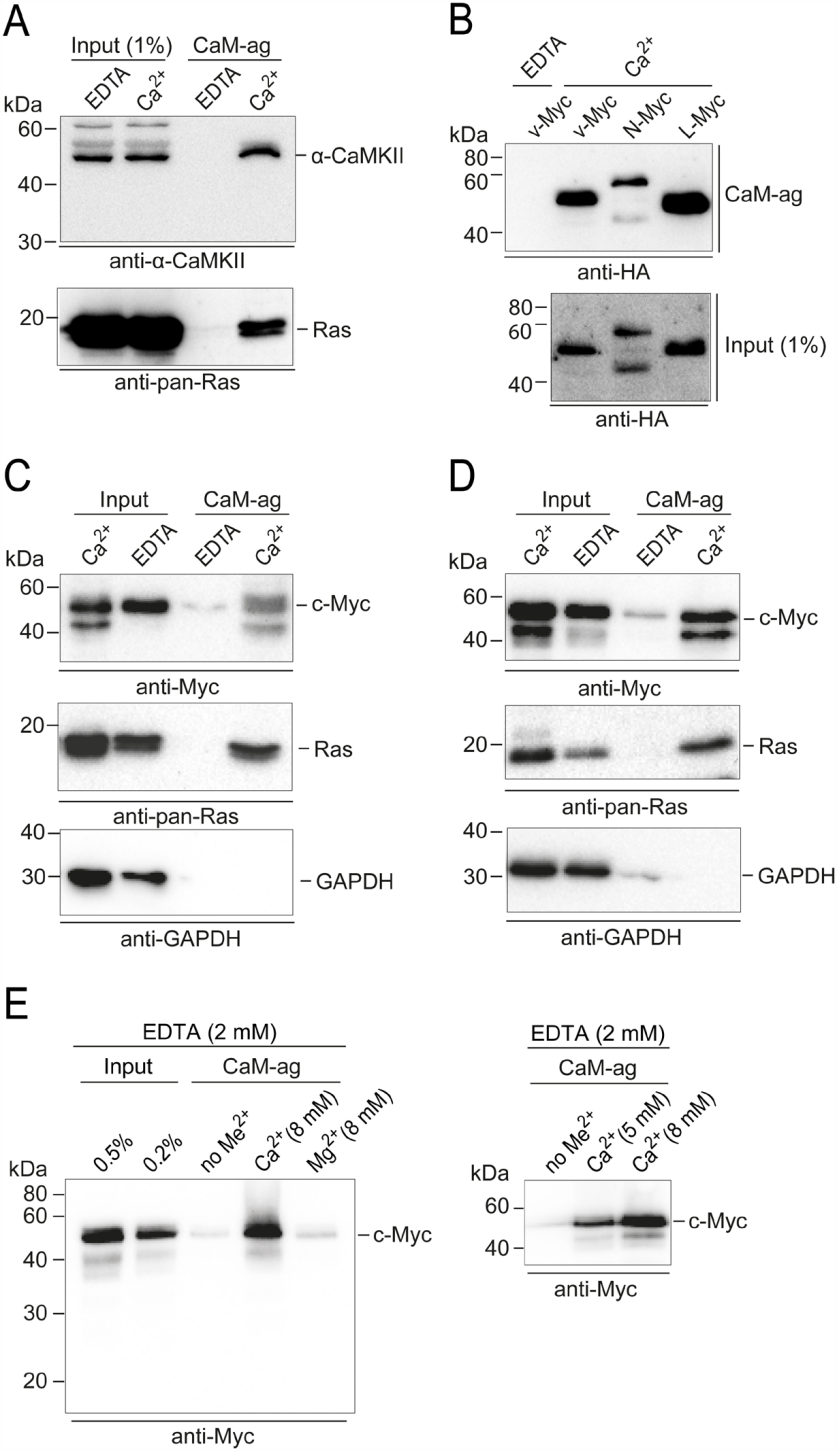
PPI of CaM and Myc family members Whole cell extracts of distinct cell lines and tissues were used in CaM-agarose (CaM-ag) pull-down experiments. **A.** For comparison, authentic CaM binding partners were analyzed first. Endogenous CaM-dependent protein kinase from whole mouse brain extracts was detected using monoclonal antibodies directed against α-CAMKII. Ras proteins were analyzed in extracts from the human colon adenocarcinoma cell line SW480 using monoclonal anti-pan-Ras antibodies. **B.** To analyze the binding of distinct Myc-family members to CaM, HA-tagged v-Myc, N-Myc, and L-Myc proteins were overexpressed in QT6 cells. Whole cell extracts were prepared and subjected to CaM-ag binding assays. Proteins eluted from the affinity matrix (*upper panel*) and aliquots of whole cell extracts (*lower panel*) were analyzed by immunoblotting using HA-specific antibodies. **C.** Endogenous c-Myc proteins were analyzed in CaM-ag binding assays using extracts from SW480 cells and monoclonal anti-c-Myc antibodies. For direct comparison, Ras proteins were analyzed from the same extracts using monoclonal anti-pan-Ras antibodies. GAPDH was used as a negative control, using monoclonal anti-GAPDH antibodies. Input: 0.25% (Ca^2+^), or 0.1% (EDTA). **D.** Analyses as in (C) of endogenous c-Myc proteins from the human embryonic kidney cell line HEK293. All binding assays were done in the presence of 2 mM EDTA, or 0.5 mM CaCl_2_ (Ca^2+^). **E.** *Left panel*: CaM-ag binding analysis of endogenous c-Myc proteins from HEK293 cell extracts containing 2 mM EDTA and supplemented either with no metal ions (no Me^2+^), or an excess (8 mM) of Ca^2+^ or Mg^2+^, respectively. *Right panel*: Analysis as before with variation of the excess Ca^2+^ concentration.

For comparison, we first confirmed the interaction of authentic binding partners of CaM [15] by the CaM-agarose pull-down assay, like CaM-dependent protein kinase from mouse brain extracts or Ras GTPases from extracts of the human colon adenocarcinoma line SW480 (Figure 4A). We then analyzed the binding of distinct Myc family members to CaM. Overexpressed HA-tagged v-Myc, N-Myc, and L-Myc proteins from QT6 cell extracts showed Ca^2+^-dependent binding to CaM-agarose with similar efficiencies (Figure 4B). In order to prove that the observed interaction of CaM with Myc is not limited to overexpressed or recombinant proteins (Figures 1 – 3), we also analyzed the interaction with endogenous c-Myc proteins in the SW480 cell line (Figure 4C) or the human embryonic kidney line HEK293 (Figure 4D). Using extracts from both cell types, Ca^2+^-dependent binding of c-Myc to CaM-agarose was observed, with similar affinity as that of the authentic CaM-binding protein Ras analyzed in parallel. No binding of GAPDH used as a control was detected (Figure 4C and 4D). To test for the metal ion specificity of the interaction, we used HEK293 cell extracts containing 2 mM EDTA throughout and supplemented with an excess (8 mM) of Ca^2+^ or Mg^2+^, respectively. Only the calcium ion containing samples led to significant binding of c-Myc to the CaM-agarose matrix (Figure 4E). Variation of the excess Ca^2+^ concentration directly corroborated the Ca^2+^ dependence of the interaction (Figure 4E).

### Analyses of Myc:CaM interactions by NMR spectroscopy

**Figure 5:**
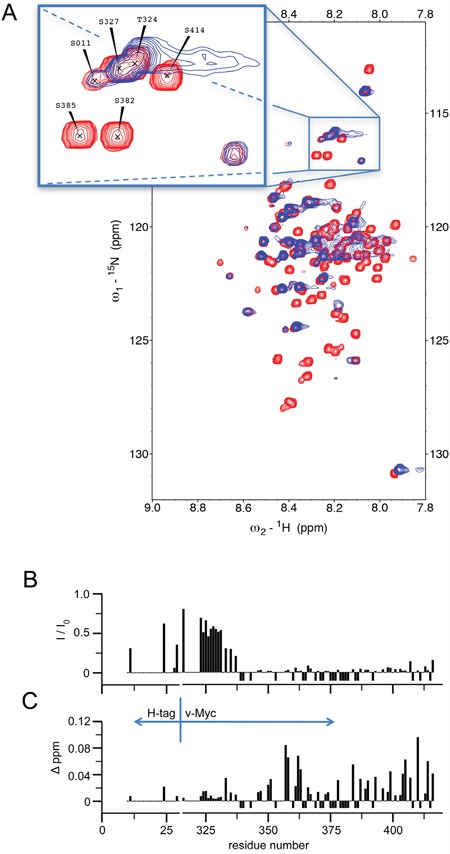
^1^H-^15^N HSQC spectra of ^15^N Myc upon addition of ^14^N CaM (1:1) **A.** Overlay of HSQC spectra recorded with ^15^N Myc free in solution (red) and with equal amounts of Myc and CaM (blue). The zoomed inlet shows a section of the spectrum where peak overlap was relatively low. All measurements were carried out with an excess of Ca^2+^ over calmodulin. **B.** The intensity ratio between the free and CaM-bound form of ^15^N Myc peaks is plotted according to the residue position (I: peak intensity of ^15^N Myc with ^14^N calmodulin present, I_0_: peak intensity of ^15^N Myc free in solution). **C.** The change in the observed peak position upon addition of ^14^N CaM to ^15^N Myc is plotted according to the residue position. Given values correspond to a pseudo ^1^H shift (measured ^1^H shift + (1/5)*^15^N shift) in order not to bias towards mainly ^15^N shifting residues. Negative values in either plot indicate that the intensity of an assigned peak position went below noise level upon addition of CaM; positions not assigned or overlapping in the free form were not used and have 0-values. All measurements were carried out with excess Ca^2+^.

Interaction between Myc and CaM was additionally probed using nuclear magnetic resonance (NMR) spectroscopy. 2D ^15^N-^1^H HSQC spectra were recorded for ^15^N-labeled Myc in the apo-state and when bound to unlabeled ^14^N-CaM. Figure 5A shows an overlay of HSQC spectra of apo Myc and after binding to CaM. The narrow chemical shift distribution obtained for Myc is typical for an intrinsically disordered protein (IDP) and results from the sizeable conformational flexibility of Myc in its apo-state. Binding to CaM led to significant changes in the NMR spectra of the bound state where most of the peaks have disappeared, while the positions of the remaining peaks were largely unchanged. The disappearance of peaks is due to the fact that residues of the IDP which are located in the protein interaction site display significant reduction of their residual conformational flexibility. Their motional behavior is thus governed by the overall tumbling of the protein complex. In contrast, Myc residues that are not involved in the interaction with CaM retained their pronounced intramolecular flexibility and could still be observed in the bound state. Due to their conformational averaging and the absence of intermolecular interactions with CaM, their chemical shifts were largely unaffected. It should be noted, however, that the overall tumbling of the Myc:CaM complex is slowed down by the flexible parts of Myc, as they exist as extended polypeptide segments and thus lead to an increase of the overall correlation time.

Residue plots of the observed changes in Myc NMR signal intensity (Figure 5B) and chemical shift changes (Figure 5C) upon binding to CaM were recorded. The recombinant v-Myc protein fragment used corresponds to residues 314 through 416 of the chicken c-Myc sequence (cf. Figure 2C), encompassing the bHLH-LZ domain starting at residue 331. Residues located at the N-terminal end of this fragment were nearly unaffected by binding to CaM. In contrast, residues located beyond residue position 340 were significantly affected in the bound state. Although most of the residue signals in this segment disappeared upon binding, some residues could be detected also in the bound state. The largest chemical shift changes were observed for residues clustering around position 360 and at the C-terminus. Taken together, the NMR data indicate that a segment of the v-Myc protein confined to the bHLH-LZ domain comprises the interaction site with CaM. Although it should be noted that NMR chemical shift mapping identifies affected sites and not necessarily only ligand binding sites, it can be concluded that the NMR derived interaction site is in compelling agreement with the biochemical mapping. The region beyond residue 340 and extending across position 360 (Figure 5B, 5C) of the chicken c-Myc sequence corresponds to a region on human c-Myc encompassing the border between the deletion mutants ΔF and ΔG that do not bind CaM (cf. Figure 2). In order to rule out that the observed interactions between Myc and CaM were mainly due to non-specific electrostatic interactions, ^15^N-^1^H HSQC spectra for free and CaM-bound ^15^N-labeled Myc were also recorded in low and high salt concentrations (Supplementary Figure S1). Even under high salt conditions Myc and CaM formed a stable complex in solution, ruling out a major relevance of electrostatic contributions but pointing towards residue-specific interactions. These experiments also confirmed that tag-free Myc (Supplementary Figure S1) or His-tagged Myc (Figure 5A) yielded similar results.

We have also performed the inverse experiments employing ^15^N-labelled CaM bound to NMR-invisible ^14^N-Myc (Supplementary Figure S2A). Again, there were detectable changes in the ^15^N-CaM NMR spectra which provided further experimental evidence for the formation of the Myc:CaM protein complex. In this case, however, the location of the binding site was not as straightforward. Since CaM is a well-structured protein in solution lacking significant intramolecular flexibility, the binding of extended Myc significantly increased the hydrodynamic radius of the complex and therefore all residues in CaM experienced a similar change in NMR spin relaxation. The protein interaction thus led to a general global reduction of signal intensities for CaM residues. Nevertheless, despite the small number of remaining peaks in the Myc:CaM complex, the position of disappearing residues could be used to map the Myc binding site on CaM (Supplementary Figure S2B and S2C). However, depicting the disappearing residues on the structure of CaM, the effect appeared to be present in both lobes without a clear clustering, with a slight preference towards the cores of the lobes facing away from the Ca^2+^ binding site. The NMR data would be compatible with a canonical CaM binding mode employing α-helical interaction domains on Myc interacting between the two modules of CaM, each containing two Ca^2+^-binding sites (Supplementary Figure S3). Further NMR experiments are currently performed to reveal the stoichiometry of the complex (1:1, or 2:2) and more details about the 3D structure.

### Subcellular distribution of Myc:CaM interactions

**Figure 6:**
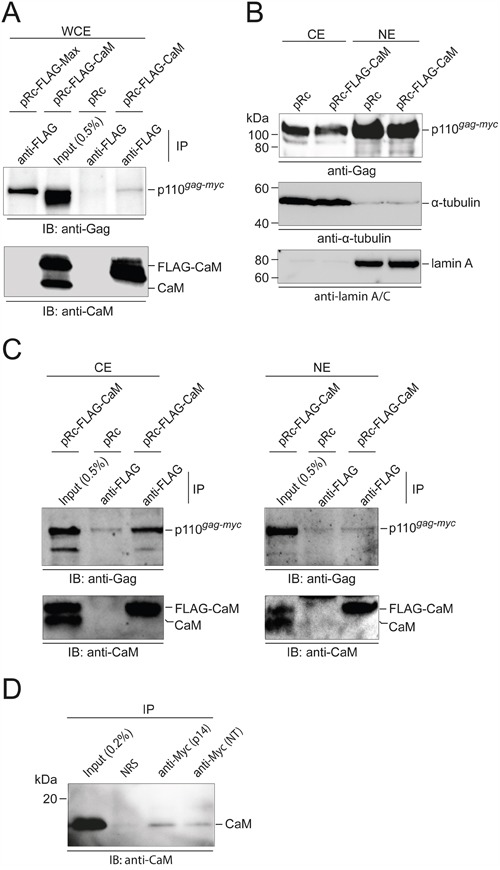
Subcellular localization of Myc:CaM interactions **A.** Co-immunoprecipitation (IP) using whole cell extracts (WCE) from Q8 cells transfected with empty vector (pRc) or pRc vectors carrying the coding regions of FLAG-tagged Max or CaM. IPs of FLAG-tagged proteins were performed in the presence of 0.2 mM CaCl_2_. Immunocomplexes were subjected to SDS-PAGE and immunoblotting (IB) with Gag-specific antiserum (*upper panel*). Precipitation efficiency of FLAG-tagged CaM was controlled using a monoclonal anti-CaM antibody (*lower panel*). **B.** Q8 cells transfected with the indicated expression vectors were subjected to biochemical cell fractionation. The distribution of the 110-kDa Gag-Myc hybrid protein, and of cytoplasmic (α-tubulin) and nuclear (lamin A) marker proteins in the cytoplasmic (CE) and nuclear (NE) extracts was detected by IB. **C.** IPs of FLAG-tagged proteins from the cytoplasmic or nuclear fractions of Q8 cells as in (B) were performed in the presence of 0.2 mM CaCl_2_. Immunocomplexes were subjected to SDS-PAGE and IB with Gag-specific antiserum (*upper panel*). Precipitation efficiency of FLAG-tagged CaM was controlled using a monoclonal anti-CaM antibody (*lower panel*). **D.** Detection of endogenous CaM in complex with v-Myc. Whole cell extracts from QEF/Rc-Myc cells were subjected to IPs using rabbit antisera directed against the carboxyl terminus (p14) or the amino terminus (NT) of v-Myc in the presence of 0.2 mM CaCl_2_. Normal rabbit serum (NRS) was used as control. Immunocomplexes were subjected to SDS-PAGE and IB using anti-CaM antibodies.

We also used co-immunoprecipitation (Co-IP) to provide evidence for Myc:CaM PPI and to determine their subcellular localization. Whole cell extracts from Q8 cells transfected with vectors expressing FLAG-tagged Max or CaM were used in the Co-IP. In confirmation of the GST-CaM and CaM-agarose pull-down experiments, the p110 Gag-Myc hybrid protein encoded by MC29 was co-precipitated both by the Max control and by CaM (Figure 6A). Q8 cells were then subjected to biochemical cell fractionation. Direct immunoblotting confirmed that the Gag-Myc hybrid protein was found mainly in the nuclear fraction, but substantial amounts were also present in the cytoplasmic fraction (Figure 6B). CaM was mainly located in the cytoplasm, but also in the nuclear fraction (Supplementary Figure S4). Co-IP analyses revealed that the Myc:CaM PPI occurred predominantly in the cytoplasm (Figure 6C). Co-IP analyses using two independent Myc sera (11) were performed to confirm complex formation of endogenous CaM with v-Myc proteins (Figure 6D).

### Functional aspects of Myc:CaM interactions

**Figure 7:**
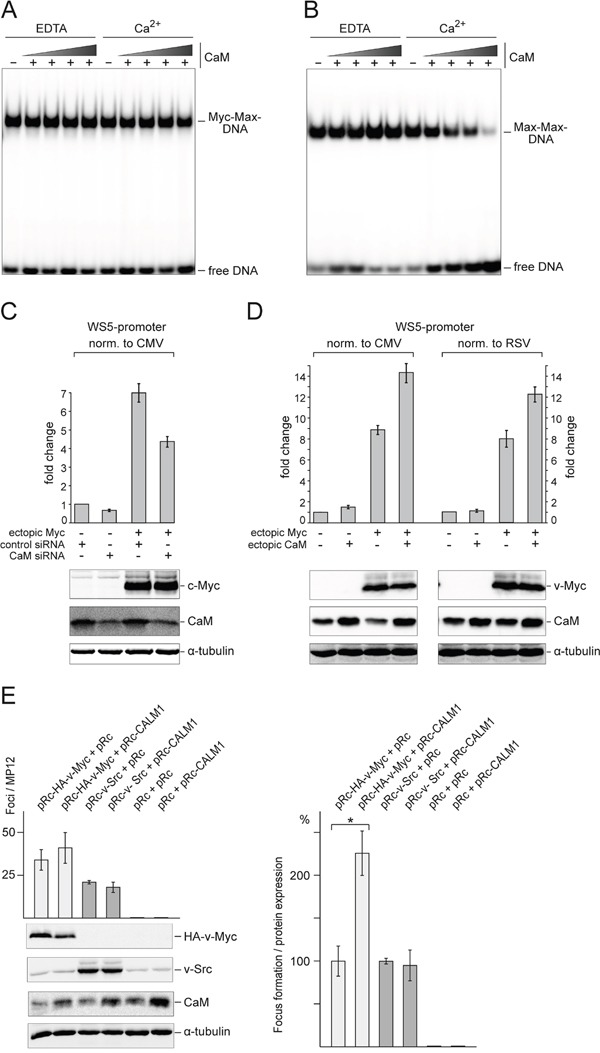
Effect of CaM on DNA binding, transcriptional activity, and transforming potential of Myc **A.** For EMSA analysis, recombinant Myc:Max protein complex (1 nM) was incubated with increasing amounts (62.5, 125, 250, or 500 nM) of CaM in the presence of CaCl_2_ (1 mM) or EDTA (2 mM) for 30 min at RT. After addition of 0.1 ng [^32^P]-radiolabeled double-stranded DNA (18-mer containing a CACGTG motif), the reactions were incubated for further 30 min at RT followed by native PAGE (6% wt/vol) and visualization by phosphor imaging. **B.** Recombinant Max protein (5 nM) was used to perform EMSA experiments as described in *A*. **C.** The effect of CaM on Myc transcriptional activity was analyzed using siRNAs targeting *CALM1* and *CALM2* transcripts. Reporter constructs pGL3-WS5 (expression of *Firefly* luciferase controlled by the Myc target gene promoter WS5) and pcDNA3.1-*R*luc (expression of *Renilla* luciferase controlled by the CMV promoter) were co-transfected into QT6 cells together with empty pcDNA3.1 or pcDNA3.1-HA-c-Myc and control siRNA or siRNA directed against CALM transcripts. 48 h after transfection, luciferase activities were measured and *Firefly* luciferase activities were normalized to *Renilla* luciferase activities. The fold changes calculated from three independent experiments (± SD) are shown. Protein expression was monitored by immunoblotting using antibodies directed against HA-tag (for HA-c-Myc), CaM, and α-tubulin. **D.** Effect of CaM overexpression on Myc transcriptional activity. Reporter constructs pGL3-WS5 and pcDNA3.1-*R*luc or pRc-*R*luc (expression of *Renilla* luciferase controlled by RSV-LTR) were co-transfected into QT6 cells together with empty pRc or pRc-HA-v-Myc and empty pcDNA3.1 or pcDNA3.1-CaM. 48 h after transfection, luciferase activities were measured and *Firefly* luciferase activities were normalized to *Renilla* luciferase activities. The fold changes calculated from four independent experiments (± SD) are shown. Protein expression was monitored by immunoblotting using antibodies directed against HA-tag (for HA-v-Myc), CaM, and α-tubulin. **E.** Effect of CaM overexpression on v-Myc-induced cell transformation. *Left panel*: QEF were cotransfected in triplicate on MP12 dishes with 1-μg aliquots of the plasmids pRc-HA-v-Myc, pRc-v-Src, pRc-CALM1, or the empty pRc vector as indicated. Cells were kept under agar overlay and foci were scored after 3 weeks. Proteins from equal amounts of cell extracts prepared 1 day after transfection were analyzed by immunoblotting using specific antisera directed against the HA tag of v-Myc, or against v-Src, CaM (CALM), and α-tubulin (TUBA). *Right panel*: Because of differences in ectopic Myc expression levels, focus counts from n=3 independent experiments were normalized to oncoprotein expression quantified by densitometry. Statistical significance was assessed by using a paired Student t test (*P = 0.014).

We first tested for any possible effects of CaM on *in vitro* DNA binding by Myc:Max heterodimers or Max homodimers. Interestingly, there was no effect on Myc:Max DNA binding either in the presence or absence of Ca^2+^ (Figure 7A), whereas DNA binding of Max homodimers was inhibited by increasing CaM concentrations in the presence of Ca^2+^ (Figure 7B). It should be noted, that Myc:Max:DNA complexes have very low dissociation constants and are significantly more stable than Max:Max:DNA complexes [24]. Multimeric complex formation of the added CaM with Myc:Max:DNA or Max:Max:DNA could be ruled out since no band shifts were observed in the EMSA analyses upon addition of CaM (Figure 7A and 7B).

We also analyzed any possible effects of CaM on Myc transcriptional activity, using reporter constructs containing the promoter of the Myc target gene *WS5* [26]. Reduction of CaM protein levels by siRNA-mediated suppression of *CALM1* and *CALM2* gene expression led to a decrease of the *WS5* reporter gene transcription normalized to a control promoter (Figure 7C). On the other hand, ectopic CaM overexpression led to an increase of reporter gene transcription under control of the Myc target gene promoter, normalized to two different control promoters (Figure 7D).

To test if overexpressed CaM has any effect on the oncogenic potential of v-Myc, focus formation of primary QEF was assayed by cotransfection of plasmids encoding v-Myc or – for comparison – v-Src, and CaM. Expression of ectopic CaM in addition to the ubiquitous endogenous protein led to a moderate but consistent increase in the number of foci induced by v-Myc, whereas no effect of CaM on v-Src-triggered transformation was observed (Figure 7E). No foci were generated by cells transfected with the CaM expression vector alone. Quantification of focus formation normalized to v-Myc or v-Src protein expression levels revealed that overexpressed CaM led to an approximately twofold increase of v-Myc-induced cell transformation, whereas v-Src-induced transformation was unaffected (Figure 7E).

The level and subcellular distribution of proteolytic cleavage products (Myc-nick) generated by calcium-dependent cytoplasmic proteases [21] were not affected by overexpression of CaM (Supplementary Figure S5). We also investigated any possible direct effects of the established CaM inhibitors trifluoperazine and W-7 [15, 27] on the Myc:CaM interaction. Both in CaM-agarose pull-down experiments and in Co-IP analyses we saw no effect of these inhibitors on the interaction between CaM and the Gag-Myc hybrid protein expressed in MC29-transformed Q8 cells (Supplementary Figure S6). This is in agreement with the previous report that commonly used CaM inhibitors had little effect on the interaction of CaM with bHLH proteins like E12 [28].

## DISCUSSION

**Figure 8:**
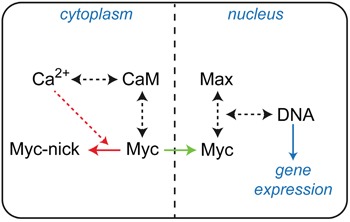
Basic diagram of interconnections between Myc and Ca^2+^/CaM signaling The binding reactions and equilibria involved are indicated by black dashed arrows, cytoplasmic proteolytic processing or nuclear transport of Myc is depicted by red or green arrows, respectively. Specific cleavage to Myc-nick requires the action of Ca^2+^-dependent proteases (red dashed arrow).

Carcinogenesis can be regarded as a consequence of pathological alterations in molecular interactions and signaling pathways. Deregulated Myc expression affects the level of Myc:Max complexes and disturbes the physiological equilibrium in the entire Myc–Max network, leading ultimately to the initiation and progression of cancer [2, 5, 6, 10, 29]. Therefore, investigations of dynamic molecular interactions are crucial in order to reveal the underlying regulatory mechanisms of complex formations in this network. It is a well-established concept in signal transduction that second messenger fluxes play a major role in altering PPIs and protein functions. Previous discoveries hinting at a possible involvement of the second messenger Ca^2+^ in Myc signaling encouraged us to analyze a putative connection to the major Ca^2+^-sensor CaM. On the one hand, the gene encoding the CaM-binding protein BASP1 has been identified as a Myc target and the BASP1 protein as an inhibitor of Myc function [11]. On the other hand, proteolytic cleavage of Myc by calpains, a family of calcium-dependent cysteine proteases, generates a cytoplasmic form of Myc (Myc-nick) with important functions in cell differentiation [21, 30]. In addition, CaM can bind to and modulate transcriptional regulators of the bHLH class like E12 and others [17–19]. Furthermore, it has been reported that Myc amplifies calcium signaling required for the stimulation of B lymphocyte proliferation and differentiation, providing evidence for converging Myc and Ca^2+^ signaling pathways [31]. Here, we have identified a direct physical interaction of Ca^2+^-loaded CaM with the oncogenic bHLH-LZ transcription factor Myc. CaM is an ubiquitous Ca^2+^-sensing receptor protein which regulates numerous fundamental cellular processes in eukaryotic cells via second messenger controlled interactions with hundreds of target proteins. Ca^2+^/CaM-dependent pathways are implicated in physiological cell cycle progression, but also in tumorigenesis [15, 16, 32]. A whole range of mitogenic factors stimulate cell growth upon activation of their respective receptors by inducing a transient increase of the intracellular concentration of free Ca^2+^ [16, 33]. Proliferative signals are primarily routed through the Ca^2+^-dependent interaction and allosteric activation of CaM-dependent kinases and phosphatases [15, 16]. However, numerous non-kinase/phosphatase interaction partners of CaM have also been identified [34]. A very interesting example is the highly specific interaction of CaM with oncogenic K-Ras, a member of the Ras family of oncoproteins [35, 36]. This interaction leads to a suppression of non-canonical Wnt/Ca^2+^ signaling by a reduction of CaMKII activity and contributes substantially to the oncogenic properties of K-Ras [36]. In this report, we provide evidence for a direct link between CaM and an oncogenic transcriptional regulator. Myc has a pivotal role in nearly all fundamental cellular functions, many of which are also regulated by Ca^2+^-signaling, and in deregulated form Myc is a hallmark of most human cancers. Our results support and extend available indications that Ca^2+^/CaM-mediated signaling and Myc regulatory pathways are at least partially interconnected and that possibly both cytoplasmic and nuclear functions of Myc are affected (Figure 8) [21, 30, 31]. The biochemical interaction studies and mapping analyses indicated that CaM binds with highest affinity to the free monomeric Myc protein. This is supported by the observation that Max, which in contrast to Myc readily forms homodimers in solution, did not bind CaM in the homodimeric form. The LZ region is required for efficient dimerization [22, 23], and a synthetic Max construct lacking LZ bound CaM as efficient as Myc. Based on these mapping studies, the interaction site for CaM binding was located to the bHLH region of the bHLH-LZ protein Myc. This was directly confirmed by the NMR spectroscopic analyses of Myc:CaM interactions, and is also in agreement with previous reports on the interaction of CaM with proteins of the bHLH class like the E-proteins E12 or E47 [17–19]. It has been proposed that the mechanism of interaction between CaM and the bHLH E-proteins is different from the canonical molecular recognition of target enzyme peptides by CaM, and that this could explain the observed insensitivity of this interaction towards standard CaM inhibitors [28]. Notably, the CaM:Myc interaction as measured in pull-down and CoIP assays was also not affected by these inhibitors. This could indeed point to a non-canonical binding mode of CaM to Myc, but alternatively could be due to stronger binding affinities of Myc. It was also reported that Ca^2+^-signaling regulates myogenesis by CaM-mediated inhibition of DNA binding by E-protein homodimers, thereby promoting DNA-binding by bHLH heterodimers of the MyoD and E-protein families [19]. In an interesting coincidence with these previous reports [17, 19], DNA binding by Max homodimers, but not by the thermodynamically more stable Myc:Max heterodimers was affected by CaM in the *in vitro* EMSA assays reported here. The most interesting functional link between CaM and Myc was provided by cell-based assays measuring the specific transcriptional and cell transforming activities of Myc. The negative effect of experimental downregulation of endogenous CaM and the positive effect of overexpression of ectopic CaM on specific promoter activation of an authentic Myc target gene support the view that Ca^2+^/CaM-signaling and Myc regulatory pathways act synergistically. This is in agreement with the report on synergistic Myc and Ca^2+^ signaling pathways in B cell proliferation and differentiation [31]. Furthermore, the specific and consistent positive effect of CaM overexpression on Myc cell transforming capacity underscores the putative synergistic effect of the interaction between these key regulatory proteins. The quantitatively moderate effects upon experimental CaM overexpression both on transcriptional and transforming activity could be due to the ubiquitous presence of the endogenous CaM protein expressed in identical structure from up to three independent genes (*CALM1*, *2*, and *3*) in all eukaryotic cells. In summary, the biochemical and structural results presented here reveal that Myc and CaM directly interact with each other, and further analyses are required to elucidate in detail the molecular mechanisms of functional interconnections between Myc regulatory pathways and Ca^2+^ second messenger signaling.

## MATERIALS AND METHODS

### Chemicals

Calmodulin inhibitors W-7 and trifluoperazine (TFP), and CaM-agarose were purchased from Sigma-Aldrich. Glutathione Sepharose 4B beads and Protein A Sepahrose CL-4B beads were purchased from GE Healthcare Life Science, Ni-NTA Agarose from Qiagen. The Luciferase Assay system (Promega) was used to quantify firefly luciferase activity, benzyl-coelenterazine (NanoLight) was used as substrate for *Renilla* luciferase.

### Antisera, antibodies, and protein analyses

v-Myc-, Gag-, and Max-specific rabbit antisera were described previously [11, 26, 37]. The monoclonal mouse antibodies anti-α-tubulin and anti-FLAG were purchased from Sigma-Aldrich (T5168; F3165). Mouse monoclonal anti-CaM was purchased from Merck Millipore (05-173), anti-HA from Covance (MMS-101P), anti-α-CaMKII from Santa Cruz (sc-13141), and anti-pan-Ras from Thermo Fisher Scientific (MA1-012). Mouse monoclonal anti-lamin A/C (4777), rabbit monoclonal anti-c-Myc (13987), and rabbit monoclonal anti-GAPDH (5174) were obtained from Cell Signaling Technology. Monoclonal mouse antibodies specific for v-Src (327) were obtained from Calbiochem. SDS-PAGE, immunoblotting, and immunoprecipitation were carried out as described (11). For densitometry, relative protein expression levels were determined with the program ImageQuant TL (GE Healthcare).

### DNA constructs and siRNAs

For expression of GST fusion-proteins, coding regions of chicken *max* (codons 22 to 113), chicken *BASP1*, and chicken *CALM1* were ligated into pET42a. The bacterial expression vectors pET3d-p14*max* and pET3d-*mycmax* were described previously [24]. For preparation of 6×His-tagged recombinant v-Myc proteins, codons 314 to 416 or codons 314 to 383 (ΔLZ) of v-*myc* were PCR amplified and ligated into pET11d. The same strategy was used to generate 6×His-tagged MaxΔLZ (codons 22 to 79). Coding sequences of HA-tagged full-length chicken Max, N-Myc, L-Myc, and of viral Myc were PCR amplified and ligated into the eukaryotic expression vector pRc/RSV. DNA fragments encoding N-terminal Flag-tagged chicken Max and CaM were cloned into pRc/RSV. pRc-*R*luc, pcDNA3.1-Rluc, and pcDNA3.1-CaM were generated by ligating the coding regions of full-length humanized *Renilla* luciferase [38] or full-length chicken *CALM1* into the eukaryotic expression vectors pRc/RSV or pcDNA3.1. The reporter construct pGL3-WS5 (pLUC-WS5) containing the quail *WS5* promoter has been described [11, 37]. Expression constructs encoding HA-tagged human full-length c-Myc and scanning Myc deletion mutants (ΔA through ΔG) have been described [20, 21]. siRNA duplexes directed against quail *CALM1* (5'-GCAGAGCUACGUCAUGUUAUT/5'-UAACAUGACGUAGCUCUGCUdG) and *CALM2* (5'-GCAAUGGCACAAUUGACUUUT/5'-AAGUCAAU UGUGCCAUUGCCdA) transcripts were designed using the program BLOCK-iT (Thermo Fisher Scientific) and prepared by solid phase synthesis [39].

### Expression and purification of recombinant proteins

Recombinant Max protein and Myc-Max protein complexes were prepared as described [24]. Recombinant CaM was expressed from pET3d-CaM in *E. coli* Rosetta(DE3)pLysS for 3 h at 37°C. Cells were collected by centrifugation, resupended in buffer A (20 mM Tris-HCl pH 7.5, 80 mM NaCl, 4 mM EDTA, 1 mM DTT) and lysed at 1,300 psi using a French press. After centrifugation, the soluble fraction was heated to 52°C for 5 min and heat-denatured *E. coli* proteins were removed by centrifugation. The supernatant containing the heat-stable CaM was loaded onto a Resource™ Q anion exchange column (GE Healthcare) equilibrated in buffer A. Chromatography on an ÄKTA Purifier (GE Healthcare) was carried out by initial elution with buffer A, followed by a linear gradient from 0 to 1 M NaCl in the same buffer at a flow rate of 1 ml/min. CaM containing fractions were loaded onto a Superdex-75 gel filtration column (GE Healthcare) equilibrated with buffer B (50 mM NaH_2_PO_4_/Na_2_HPO_4_ pH 7.2, 140 mM NaCl, 1 mM EDTA, 1 mM DTT) and then eluted with the same buffer. 6×His-tagged Myc and Max proteins (pET11d-constructs) were expressed in *E. coli* Rosetta(DE3)pLysS for 3 h at 30°C. Cells were collected by centrifugation, resuspended in ice-cold binding buffer (50 mM NaH_2_PO_4_ pH 8, 300 mM NaCl, 10 mM imidazole) and lysed at 1,300 psi using a French press. Lysates were clarified by centrifugation and incubated with Ni-NTA agarose for 1 h at 4°C. Beads were collected by centrifugation and resuspended in washing buffer (50 mM NaH_2_PO_4_ pH 8, 300 mM NaCl, 20 mM imidazole), this washing procedure was repeated twice. 6×His-tagged proteins were eluted from the beads using elution buffer (50 mM NaH_2_PO_4_ pH 8, 300 mM NaCl, 500 mM imidazole). Eluted proteins were loaded onto a Superdex-75 gel filtration column (GE Healthcare) equilibrated with gel filtration buffer (30 mM Tris-HCl pH 7.5, 150 mM NaCl, 0.5 mM DTT) and then eluted with the same buffer. GST-fusion proteins (pET42a-constructs) were expressed in *E. coli* Rosetta(DE3)pLysS for 3 h at 30°C. Bacterial lysates were prepared as described above and clarified lysates were incubated with glutathione-sepharose beads for 1 h at 4°C. Beads were washed using GST-lysis buffer (30 mM Tris-HCl pH 7.5, 200 mM NaCl, 5% glycerol, 0.5% Triton-X100) and GST-fusion proteins bound to the beads were used for pull-down experiments or stored at -80°C.

### Electrophoretic mobility shift assay (EMSA)

Protein-DNA binding reactions were performed as described [24, 37] under conditions similar to those described for the analysis of bHLH proteins [17]. DNA-binding proteins were pre-incubated with CaM for 30 min at 25°C, followed by the addition of the DNA-probe and incubation for another 30 min. Protein-DNA complexes were resolved by native 6% (wt/vol) polyacrylamide gel electrophoresis (PAGE), and radioactive signals were quantified using a PhosphorImager (GE Healthcare).

### Cell culture

Normal quail embryo fibroblasts (QEF) and the established quail cell lines Q8, QEF/MC29, QEF/RCAS-HA-v-Myc, and QEF/Rc-Myc were grown as previously described [11, 26, 37, 40]. QT6 cells are a line of chemically transformed QEF with normal c-*myc* expression levels [26, 40]. DNA transfection of quail cells was carried out using the calcium-phosphate method. Human colon carcinoma cells SW480 and human embryonic kidney cells HEK293 were grown in DMEM supplemented with 10% (vol/vol) FBS.

For the preparation of whole cell extracts, cells were scraped from the dishes and washed with PBS. After centrifugation the cell pellet was resuspended in ice-cold lysis buffer (30 mM Tris-HCl pH 7.5, 150 mM NaCl, 5% glycerol, 0.5% Triton-X100, and 0.5 mM CaCl_2_ or 2 mM EDTA; protease inhibitors: 2 μg/ml aprotinin, 1 μg/ml leupeptin, 1 μg/ml pepstatin A). For biochemical cell fractionation, cells were resuspended in the hypotonic extraction buffer A (10 mM HEPES pH 7.9, 1.5 mM MgCl_2_, 10 mM KCl, 1 mM DTT; protease inhibitors: 1 mM PMSF, 2 μg/ml aprotinin, 1 μg/ml leupeptin, 1 μg/ml pepstatin A) and plasma membranes were mechanically disrupted using a Dounce homogenizer. The cytoplasmic fraction was separated from cell nuclei by centrifugation, and precipitated nuclei were washed twice using extraction buffer A. Nuclear proteins were extracted using the high-salt extraction buffer B (10 mM HEPES pH 7.9, 5% glycerol, 420 mM NaCl, 1 mM DTT, 0.5 mM CaCl_2_, 0.5% Triton X-100; protease inhibitors: 2 μg/ml aprotinin, 1 μg/ml leupeptin, 1 μg/ml pepstatin A). To lower the salt concentration for immunoprecipitation experiments, nuclear extracts were diluted using an equal volume of extraction buffer C (10 mM HEPES pH 7.9, 5% glycerol, 1 mM DTT, 0.5 mM CaCl_2_, 0.5% Triton X-100; protease inhibitors: 2 μg/ml aprotinin, 1 μg/ml leupeptin, 1 μg/ml pepstatin A). The cytoplasmic fraction was adjusted to 150 mM NaCl, 5% glycerol, and 0.5% Triton-X100. For preparation of whole mouse brain extracts, the brain was washed in ice-cold PBS and homogenized with a spatula. After the addition of ice-cold lysis buffer (30 mM Tris-HCl pH 7.5, 150 mM NaCl, 5% glycerol, 0.5% Triton-X100, and 0.5 mM CaCl_2_ or 2 mM EDTA; protease inhibitors: 2 μg/ml aprotinin, 1 μg/ml leupeptin, 1 μg/ml pepstatin A) the tissue was further disrupted using a Dounce homogenizer. The lysate was clarified by centrifugation.

Calcium-phosphate-mediated DNA transfection and quantification of cell transformation by focus formation were performed as described (11, 40). The constructs pRc-HA-v-Myc and pRc-v-Src have been described (11, 40). pRc-CALM1 was generated by ligating the coding region of the chicken *CALM1* gene into the eukaryotic expression vector pRc/RSV.

### Protein-protein interaction analyses

For pull-down experiments, GST-fusion proteins on glutathione sepharose beads or CaM-agarose (CaM-ag) beads were incubated with cell extracts or recombinant proteins for 2 h at 4°C. To avoid unspecific binding of recombinant prey proteins to GST bait proteins, BSA was added to the binding buffer to a final concentration of 50 μg/ml. After binding, beads were washed four times with GST-lysis buffer. Proteins were denatured by adding Laemmli buffer and subjected to SDS-PAGE and immunoblotting experiments. For immunoprecipitation (IP), clarified cell extracts were incubated with anti-Flag antibodies (2 μg per sample) and incubated on ice for 1 h. After addition of Protein-A-sepharose CL-4B (GE Healthcare), samples were incubated for 2 h at 4°C. Immunocomplexes were washed four times with lysis buffer (30 mM Tris-HCl pH 7.5, 150 mM NaCl, 5% glycerol, 0.5% Triton-X100, and 0.2 mM CaCl_2_; protease inhibitors: 2 μg/ml aprotinin, 1 μg/ml leupeptin, 1 μg/ml pepstatin A). After addition of Laemmli buffer, IP samples were subjected to SDS-PAGE and immunoblotting.

Metabolic labeling of cells with [^35^S]-methionine and preparation of cell lysates under native conditions using NDL buffer w/o EDTA (20 mM Tris HCl pH 8.0, 200 mM LiCl, 0.5% (v/v) Igepal CA-630; protease inhibitors: 2 μg/ml aprotinin, 1 μg/ml leupeptin, 1 μg/ml pepstatin A) has been described (11). For protein pull-down, 500-μl aliquots (6 x 10^7^ c.p.m.) of lysate were supplemented with CaCl_2_ or EDTA to final concentrations of 0.5 mM or 1.0 mM, respectively. 40-μl aliquots of CaM-ag bead suspension (Sigma-Aldrich) were added to the lysates and incubated for 2 h at 4°C on an overhead shaker. Beads were washed four times with NDL buffer supplemented with CaCl_2_ or EDTA, then suspended in 100 μl of boiling buffer (10 mM sodium phosphate pH 7.2, 0.5% (w/v) SDS, 2 μg/ml aprotinin), followed by heating to 100°C for 2 min. After addition of 400 μl dilution buffer (10 mM sodium phosphate pH 7.2, 187.5 mM NaCl, 1.25% (v/v) Igepal CA-630, 1.25% (w/v) sodium deoxycholate, 2 μg/ml aprotinin), beads were pelleted, and the supernatant subjected to IP as described (11) using Protein-A-sepharose. Proteins were separated by 10% (w/v) SDS-PAGE, and ^35^S-emitted radiation detected by fluorography using a Typhoon FLA 7000 bioimager (GE Healthcare) or X-ray films.

### Transactivation analysis

Reporter constructs pGL3-WS5 (*Firefly* luciferase) and pcDNA3.1-Rluc or pRc-Rluc (*Renilla* luciferase) were co-transfected into QT6 cells together with Myc-expression vectors (pcDNA3.1-HA-c-Myc or pRc-HA-v-Myc) and siRNAs directed against CaM or the expression vector pcDNA3.1-CaM. 48 h after transfection *Firefly* luciferase activities were determined as described [11, 37]. *Renilla* luciferase activities were quantified as described [40].

### NMR spectroscopy of recombinant Myc and CaM proteins

All experiments shown were performed with a recombinant fragment containing the v-Myc bHLH-LZ motif. As the assignment of this 131-amino acid construct was carried out with the histidine-tag present, the N-terminal residues were numbered M1 through V28 and are not Myc-derived. The following residues, S314 through A416, encompass the bHLH-LZ motif of v-Myc corresponding to the C-terminal region of chicken c-Myc (cf. Figure 2C) and were numbered accordingly [24]. The histidine-tag was retained to enhance solubility of the protein for the NMR measurements. Myc was expressed and purified as described previously [41]. Briefly, the histidine-tagged protein was expressed in E. coli using a pETM11 expression vector. M9 medium with ^15^N ammonium chloride was used for labeled expression, while LB broth was utilized for unlabeled protein production. Induction with 0.8 mM of IPTG led to accumulation of inclusion-bodies containing the protein. Following cell disruption, the inclusion bodies were washed in several steps, before the protein was resuspended in buffer containing 8 M urea to denature the protein and dissolve the inclusion bodies. The protein was purified in 8 M urea using its histidine-tag on a HisTrap affinity column (GE Healthcare). Following refolding by stepwise dialysis into measurement buffer, protein purity was verified by SDS-PAGE. For the expression of recombinant CaM, E. coli T7 bacteria containing the pETM11-CaM construct were grown in M9 medium containing ^15^N ammonium chloride or in LB broth. The expression was induced with IPTG, and cells were harvested after incubation for 4 hr at 37°C. Following lysis and lysate purification by centrifugation and filtering, the histidine-tag was used for purification on a HisTrap affinity column (GE Healthcare). Under dialysis to remove imidazole, the tag was cleaved using TEV-protease. Any uncleaved protein as well as the cleaved tag, the protease, and possible unspecific binding compounds were removed by running the sample through a HisTrap column a second time. Size exclusion chromatography was performed for the cleaved CaM, and the purity was verified by SDS-PAGE.

NMR measurements were carried out on a Varian Innova Spectrometer operating at 800 MHz. ^15^N-labeled proteins were used at a concentration of 200 μM, while the ^14^N binding partner was added at the indicated stoichiometries. Measurements were carried out in 20 mM BisTris, 50 mM NaCl, pH 6.0 at 25°C. All samples contained 10% D_2_O as lock solvent and excess Ca^2+^ to ensure that CaM was in the holo form. The 2D ^1^H-^15^N HSQC spectra were recorded with PFG enhancement [42]. All spectra were processed using NMRPipe [43] and SPARKY [44]. Relative intensities were calculated by simple division of peak intensity values generated by SPARKY, estimating volumes did not lead to significant changes in the obtained ratios. Shift values given are “pseudo-proton” as the contribution from nitrogen values was divided by a number of 5, thereby ensuring roughly equal weighting in comparison to the spectral resolution, as diagonal shifts in the spectra have about 5 x higher contribution in ^15^N shifts (ppm) as compared to ^1^H (ppm).

## SUPPLEMENTARY DATA AND FIGURES



## References

[1] Bister K (2015). Discovery of oncogenes: The advent of molecular cancer research. Proc. Natl. Acad. Sci. USA.

[2] Vogt PK (2012). Retroviral oncogenes: a historical primer. Nat. Rev. Cancer.

[3] Bister K, Jansen HW (1986). Oncogenes in retroviruses and cells: Biochemistry and molecular genetics. Adv. Cancer Res.

[4] Dalla-Favera R, Bregni M, Erikson J, Patterson D, Gallo RC, Croce CM (1982). Human c-myc onc gene is located on the region of chromosome 8 that is translocated in Burkitt lymphoma cells. Proc. Natl. Acad. Sci. USA.

[5] Stine ZE, Walton ZE, Altman BJ, Hsieh AL, Dang CV (2015). MYC, metabolism, and cancer. Cancer Discov.

[6] Dang CV (2012). MYC on the path to cancer. Cell.

[7] Bister K, Hayman MJ, Vogt PK (1977). Defectiveness of avian myelocytomatosis virus MC29: Isolation of long-term nonproducer cultures and analysis of virus-specific polypeptide synthesis. Virology.

[8] Blackwood EM, Eisenman RN (1991). Max: A helix-loop-helix zipper protein that forms a sequence-specific DNA-binding complex with Myc. Science.

[9] Eisenman RN (2001). Deconstructing Myc. Genes Dev.

[10] Conacci-Sorrell M, McFerrin L, Eisenman RN (2014). An overview of MYC and its interactome. Cold Spring Harb. Perspect. Med.

[11] Hartl M, Nist A, Khan MI, Valovka T, Bister K (2009). Inhibition of Myc-induced cell transformation by brain acid-soluble protein 1 (BASP1). Proc. Natl. Acad. Sci. USA.

[12] Maekawa S, Maekawa M, Hattori S, Nakamura S (1993). Purification and molecular cloning of a novel acidic calmodulin binding protein from rat brain. J. Biol. Chem.

[13] Green LM, Wagner KJ, Campbell HA, Addison K, Roberts SGE (2009). Dynamic interaction between WT1 and BASP1 in transcriptional regulation during differentiation. Nucleic Acids Res.

[14] Matsubara M, Nakatsu T, Kato H, Taniguchi H (2004). Crystal structure of a myristoylated CAP-23/NAP-22 N-terminal domain complexed with Ca^2+^/calmodulin. EMBO J.

[15] Berchtold MW, Villalobo A (2014). The many faces of calmodulin in cell proliferation, programmed cell death, autophagy, and cancer. Biochim. Biophys. Acta.

[16] Kahl CR, Means AR (2003). Regulation of cell cycle progression by calcium/calmodulin-dependent pathways. Endocr. Rev.

[17] Corneliussen B, Holm M, Waltersson Y, Onions J, Hallberg B, Thornell A, Grundström T (1994). Calcium/calmodulin inhibition of basic-helix-loop-helix transcription factor domains. Nature.

[18] Saarikettu J, Sveshnikova N, Grundström T (2004). Calcium/calmodulin inhibition of transcriptional activity of E-proteins by prevention of their binding to DNA. J. Biol. Chem.

[19] Hauser J, Saarikettu J, Grundström T (2008). Calcium regulation of myogenesis by differential calmodulin inhibition of basic helix-loop-helix transcription factors. Mol. Biol. Cell.

[20] Tworkowski KA, Salghetti SE, Tansey WP (2002). Stable and unstable pools of Myc protein exist in human cells. Oncogene.

[21] Conacci-Sorrell M, Ngouenet C, Eisenman RN (2010). Myc-nick: A cytoplasmic cleavage product of Myc that promotes α-tubulin acetylation and cell differentiation. Cell.

[22] Ferré-D'Amaré AR, Prendergast GC, Ziff EB, Burley SK (1993). Recognition by Max of its cognate DNA through a dimeric b/HLH/Z domain. Nature.

[23] Brownlie P, Ceska T, Lamers M, Romier C, Stier G, Teo H, Suck D (1997). The crystal structure of an intact human Max-DNA complex: New insights into mechanisms of transcriptional control. Structure.

[24] Fieber W, Schneider ML, Matt T, Kräutler B, Konrat R, Bister K (2001). Structure, function, and dynamics of the dimerization and DNA binding domain of oncogenic transcription factor v-Myc. J. Mol. Biol.

[25] Nair SK, Burley SK (2003). X-ray structures of Myc-Max and Mad-Max recognizing DNA. Molecular bases of regulation by proto-oncogenic transcription factors. Cell.

[26] Reiter F, Hartl M, Karagiannidis AI, Bister K (2007). WS5, a direct target of oncogenic transcription factor Myc, is related to human melanoma glycoprotein genes and has oncogenic potential. Oncogene.

[27] Osawa M, Swindells MB, Tanikawa J, Tanaka T, Mase T, Furuya T, Ikura M (1998). Solution structure of calmodulin-W-7 complex: The basis of diversity in molecular recognition. J. Mol. Biol.

[28] Onions J, Hermann S, Grundström T (2000). A novel type of calmodulin interaction in the inhibition of basic helix-loop-helix transcription factors. Biochemistry.

[29] Stefan E, Hart JR, Bister K (2015). Stopping MYC in its tracks. Aging (Albany NY).

[30] Anderson S, Poudel KR, Roh-Johnson M, Brabletz T, Yu M, Borenstein-Auerbach N, Grady WN, Bai J, Moens CB, Eisenman RN, Conacci-Sorrell M (2016). MYC-nick promotes cell migration by inducing fascin expression and Cdc42 activation. Proc. Natl. Acad. Sci. USA.

[31] Habib T, Park H, Tsang M, de Alborán IM, Nicks A, Wilson L, Knoepfler PS, Andrews S, Rawlings DJ, Eisenman RN, Iritani BM (2007). Myc stimulates B lymphocyte differentiation and amplifies calcium signaling. J. Cell Biol.

[32] Halling DB, Liebeskind BJ, Hall AW, Aldrich RW (2016). Conserved properties of individual Ca^2+^ binding sites in calmodulin. Proc. Natl. Acad. Sci. USA.

[33] Rozengurt E (2007). Mitogenic signaling pathways induced by G protein-coupled receptors. J. Cell. Physiol.

[34] Shen M, Zhang N, Zheng S, Zhang WB, Zhang HM, Lu Z, Su QP, Sun Y, Ye K, Li XD (2016). Calmodulin in complex with the first IQ motif of myosin-5a functions as an intact calcium sensor. Proc. Natl. Acad. Sci. USA.

[35] Villalonga P, López-Alcalá C, Bosch M, Chiloeches A, Rocamora N, Gil J, Marais R, Marshall CJ, Bachs O, Agell N (2001). Calmodulin binds to K-Ras, but not to H- or N-Ras, and modulates its downstream signaling. Mol. Cell. Biol.

[36] Wang MT, Holderfield M, Galeas J, Delrosario R, To MD, Balmain A, McCormick F (2015). K-Ras promotes tumorigenicity through suppression of non-canonical Wnt signaling. Cell.

[37] Valovka T, Schönfeld M, Raffeiner P, Breuker K, Dunzendorfer-Matt T, Hartl M, Bister K (2013). Transcriptional control of DNA replication licensing by Myc. Sci. Rep.

[38] Stefan E, Aquin S, Berger N, Landry CR, Nyfeler B, Bouvier M, Michnick SW (2007). Quantification of dynamic protein complexes using Renilla luciferase fragment complementation applied to protein kinase A activities in vivo. Proc. Natl. Acad. Sci. USA.

[39] Santner T, Hartl M, Bister K, Micura R (2014). Efficient access to 3'-terminal azide-modified RNA for inverse click-labeling patterns. Bioconjug. Chem.

[40] Raffeiner P, Röck R, Schraffl A, Hartl M, Hart JR, Janda KD, Vogt PK, Stefan E, Bister K (2014). In vivo quantification and perturbation of Myc-Max interactions and the impact on oncogenic potential. Oncotarget.

[41] Kizilsavas G, Saxena S, Zerko S, Kozminski W, Bister K, Konrat R (2013). ^1^H, ^13^C, and ^15^N backbone and side chain resonance assignments of the C-terminal DNA binding and dimerization domain of v-Myc. Biomol. NMR Assign.

[42] Farrow NA, Muhandiram R, Singer AU, Pascal SM, Kay CM, Gish G, Shoelson SE, Pawson T, Forman-Kay JD, Kay LE (1994). Backbone dynamics of a free and phosphopeptide-complexed Src homology 2 domain studied by ^15^N NMR relaxation. Biochemistry.

[43] Delaglio F, Grzesiek S, Vuister GW, Zhu G, Pfeifer J, Bax A (1995). NMRPipe: a multidimensional spectral processing system based on UNIX pipes. J. Biomol. NMR.

[44] Goddard TD, Kneller DG (2001). SPARKY 3.

