# Supplementary material for: Calcium-dependent binding of Myc to calmodulin
Source: Oncotarget. 2016 Dec 1;8(2):3327–43. doi: 10.18632/oncotarget.13759 (PMC5356885; doi:10.18632/oncotarget.13759)
Supplement: Supplementary file 1 [file oncotarget-08-3327-s001.pdf]

Calcium-dependent binding of Myc to calmodulin

SUPPLEMENTARY DATA

NMR spectroscopy

Measurements with ^{15}N CaM were carried out in the same conditions as stated in the main text. Measurements of ^{15}N Myc shown in the Supplementary Data were carried out in slightly different buffer conditions. Here, a 50 mM phosphate buffer at pH 6.5 containing 50 mM NaCl, 1mM sodium azide, and an excess of calcium was used for measurements at 25°C. Under these conditions, the protein was soluble without the histidine tag at quantities amenable to NMR-measurements. Therefore, these measurements were carried out after cleavage of the histidine tag with TEV protease. The protease and the affinity tag were removed by a second passing of the sample through a HisTrap affinity column. Measurements were carried out on a Bruker Spectrometer operating at 600 MHz. The ^{15}N labeled protein was used at a concentration of 50-100 µM, while the ^{14}N binding partner was added at the indicated stoichiometries. Dilution effects caused by the addition of ligand or 2M NaCl to the sample were compensated in the reference sample by adding buffer or the same concentrated salt solution. All other measurement conditions and data processing are equivalent to those described in the main text.

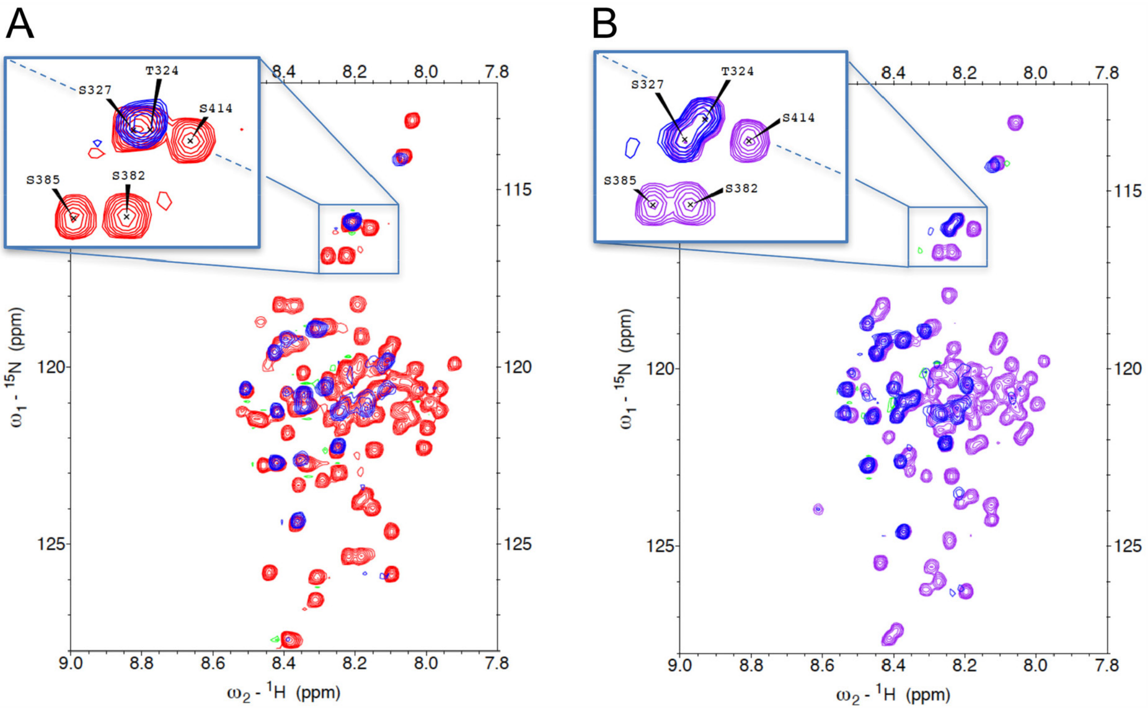

Supplementary Figure S1: ^{1}H-^{15}N HSQC spectra of tag-free Myc upon addition of CaM at low and high salt concentration. A. Overlay of HSQC spectra recorded with free ^{15}N Myc (red) and with equal amounts of Myc and CaM (blue). These measurements were carried out with the same Myc recombinant construct as in the main text, but with the histidine-tag removed and in phosphate buffer pH 6.5 containing 50 mM NaCl. The zoomed inlet shows a section of the spectrum where peak overlap is low and the relative loss in intensity for select peaks can be clearly seen. All measurements were carried out with excess Ca^{2+}. **B.** Overlay of HSQC spectra recorded with free ^{15}N Myc (purple) and with equal amounts of Myc and CaM (blue). The measurements were carried out with the same samples and conditions as in (A), but after the addition of NaCl to 500 mM. The zoomed inlet shows a section of the spectrum where peak overlap is relatively low and the maintained loss in intensity can be clearly seen. Slight signal recovery of some peaks lost upon addition of CaM can be seen, but the binding is clearly maintained even at the high salt concentration.

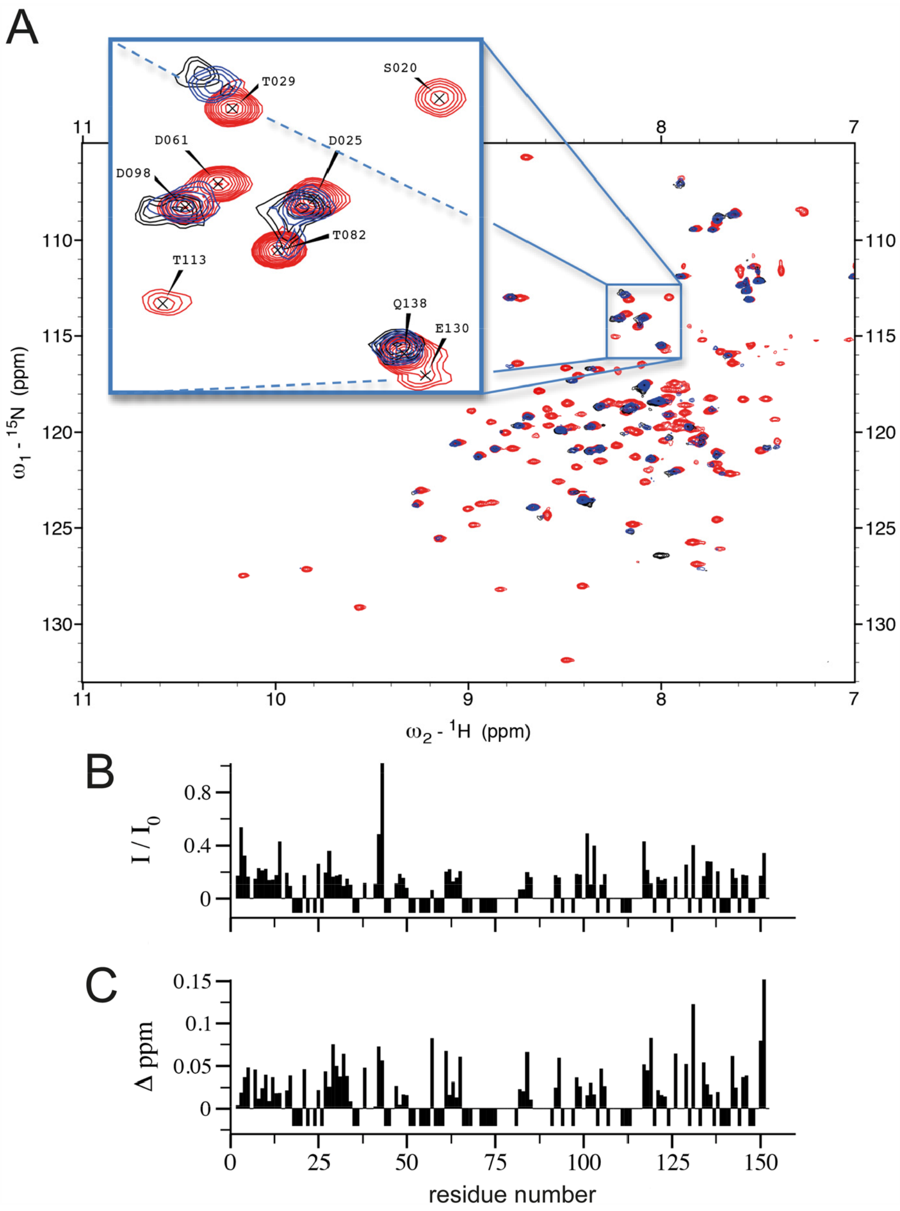

Supplementary Figure S2: ^{1}H-^{15}N HSQC spectra of CaM upon addition of Myc. A. Overlay of HSQC spectra of free ^{15}N CaM (red), and after addition of ^{14}N Myc at a 2:1 (blue) or 1:1 (black) CaM/Myc ratio. The zoomed inlet highlights the signal loss at the 1:1 ratio and demonstrates how shifts were tracked. All measurements were carried out with excess Ca^{2+}. **B.** The intensity ratio between the free and Myc-bound form of ^{15}N CaM peaks is plotted against residue position (I: peak intensity of ^{15}N CaM with ^{14}N Myc (1:1 ratio) present, I_0: peak intensity of ^{15}N CaM free in solution). **C.** Changes in peak positions upon addition of ^{14}N Myc to ^{15}N CaM are plotted. Values correspond to a pseudo ^{1}H shift (measured ^{1}H shift + (1/5)*^{15}N shift) in order not to bias towards mainly ^{15}N shifting residues. Negative values in either plot indicate that the intensity of an assigned peak position went below noise level upon addition of Myc. Positions not assigned or overlapping in the free form were not used and have 0-values.

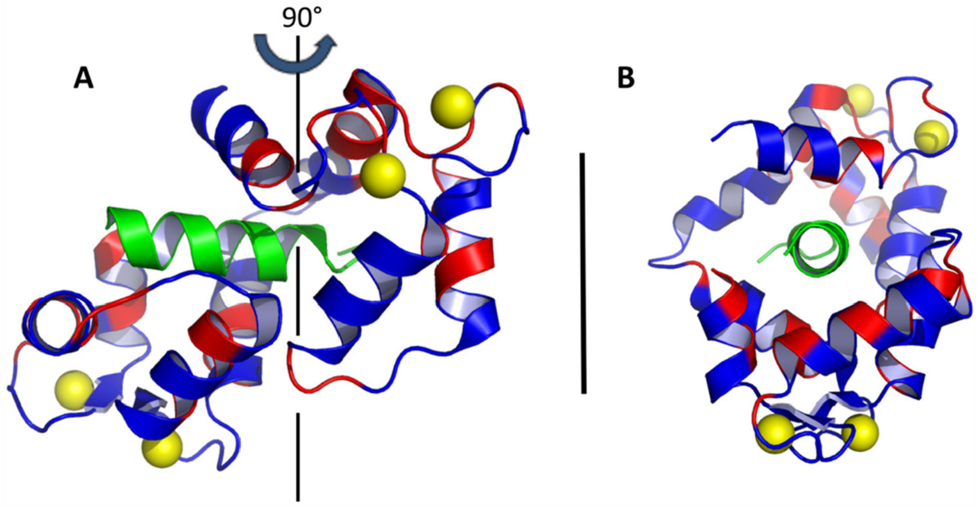

Supplementary Figure S3: Graphical representation of CaM residues affected by Myc binding. A. The coordinates of a published CaM NMR structure in complex with a connexin peptide (PDB ID: 2N6A) were used to depict the residues most affected in the NMR analysis of the CaM–Myc interaction. CaM residues are depicted in blue, assigned residues either disappearing or shifting more than 0.045 ppm (pseudo ^{1}H) in ^{15}N-^{1}H HSQC measurements upon a sub-stoichiometric addition (1:2) of ^{14}N-Myc to ^{15}N-CaM are shown in red, and the Ca^{2+} ions are depicted in yellow. The bound α-helical connexin peptide (marked in green) was not present in our measurements. **B.** View of a 90° rotation around the z-axis. The residues affected in HSQC measurements of this well-folded protein do not necessarily correlate with the binding region of Myc. However, the largest effects are found for residues in both lobes of the protein and their majority is not pointing towards the Ca^{2+}-binding sites. This is compatible with a CaM binding mode utilizing α-helical domains on Myc to interact between the two lobes of CaM, similar to the binding mode found in the interaction of the connexin peptide and CaM in this NMR structure.

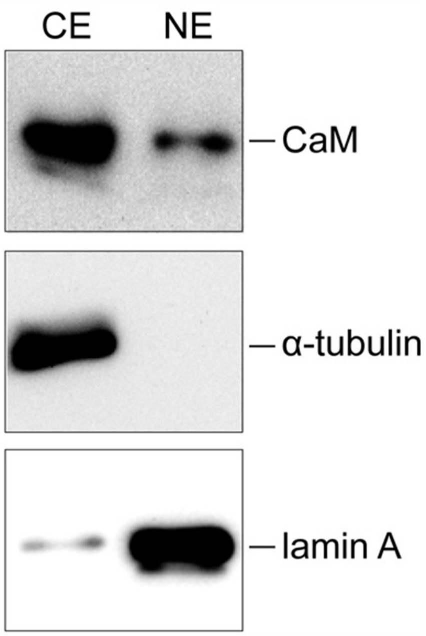

Supplementary Figure S4: Subcellular localization of CaM in v-*myc*-transformed cells. Q8 cells were subjected to biochemical fractionation. After hypotonic lysis of the plasma membrane, nuclei were washed in low-salt buffer and nuclear proteins were extracted using high-salt buffer. Proteins from cytoplasmic (CE) and nuclear (NE) extracts were separated by SDS-PAGE, and CaM and cytoplasmic (α-tubulin) or nuclear (lamin A) marker proteins were detected by immunoblotting.

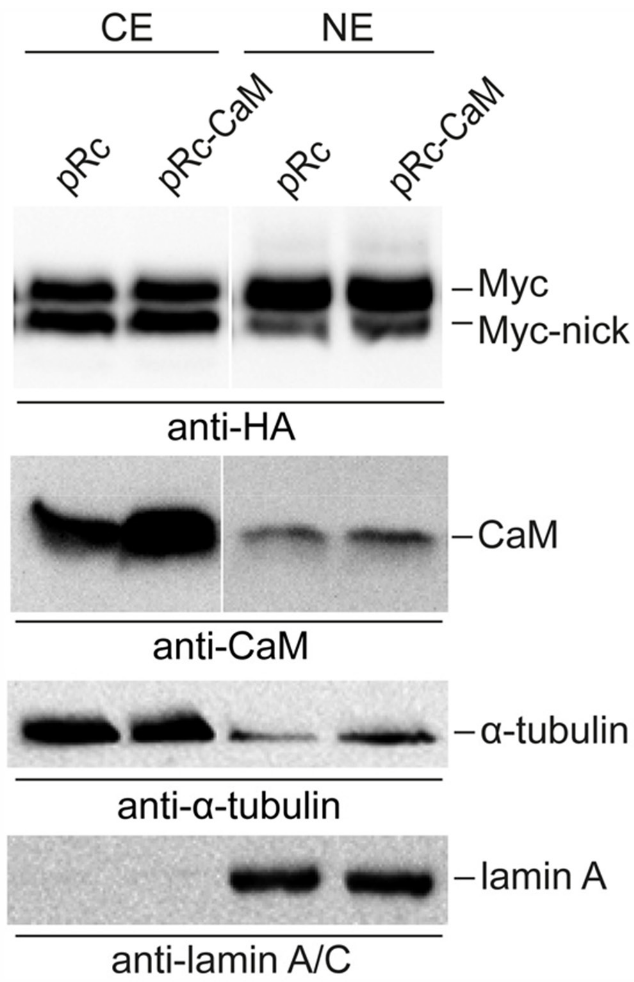

Supplementary Figure S5: Effect of CaM overexpression on Myc-nick formation and subcellular localization of Myc. Chemically transformed QT6 cells were co-transfected with pcDNA3-HA-c-Myc and pRc (empty vector) or pRc-CaM. 48 h after transfection biochemical cell fractionation was carried out and cytoplasmic (CE) and nuclear extracts (NE) were subjected to SDS-PAGE and immunoblotting using antibodies directed against the HA-epitope (Myc and Myc-nick), CaM, α-tubulin (cytoplasmic marker), or lamin A (nuclear marker).

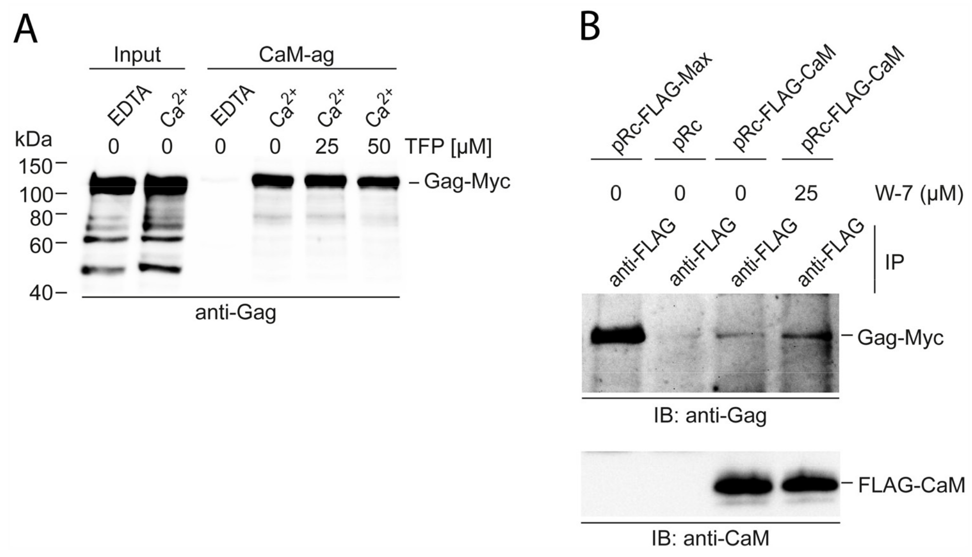

Supplementary Figure S6: Effect of CaM inhibitors on Myc:CaM complex formation. A. Whole cell extracts prepared from Q8 cells were used in calmodulin-agarose (CaM-ag) pull-down experiments in the absence or in the presence of increasing amounts of trifluoperazine (TFP). The Gag-Myc hybrid protein was detected by immunoblotting using a Gag-specific antiserum. EDTA, 2 mM; Ca^{2+}, $CaCl_2$ 0.5 mM; **B.** Co-IP using whole cell lysates prepared from Q8 cells transfected with empty vector (pRc) or pRc carrying the coding regions of FLAG-tagged Max or CaM. IPs of FLAG-tagged proteins were performed in the presence of 0.2 mM $CaCl_2$. Immunocomplexes were subjected to SDS-PAGE and immunoblotting with Gag-specific antiserum (*upper panel*). Precipitation efficiency of FLAG-tagged CaM was controlled using a monoclonal anti-CaM antibody (*lower panel*). Where indicated, the CaM-inhibitor W-7 was present in the cell culture medium for 24 h and in the cell lysate used for the Co-IP experiments.
